# (Acetyl­acetonato-κ^2^ *O*,*O*′)bis­{5-fluoro-2-[3-(4-fluoro­phen­yl)pyrazin-2-yl]phenyl-κ^2^ *N* ^1^,*C* ^1^}iridium(III)

**DOI:** 10.1107/S1600536812031546

**Published:** 2012-07-18

**Authors:** Guo-Ping Ge, Chun-Yan Li

**Affiliations:** a State Key Laboratory Base of Novel Functional Materials and Preparation Science, Institute of Solid Materials Chemistry, Faculty of Materials Science and Chemical Engineering, Ningbo University, Ningbo 315211, People’s Republic of China

## Abstract

In the title complex, [Ir(C_16_H_9_F_2_N_2_)_2_(C_5_H_7_O_2_)], the Ir^III^ atom, lying on a twofold rotation axis, is hexa­coordinated in a distorted octa­hedral geometry by two *C*,*N*-bidentate 5-fluoro-2-[3-(4-fluoro­phen­yl)pyrazin-2-yl]phenyl ligands and one *O*,*O*′-bidentate acetyl­acetonate ligand. The dihedral angles between the benzene rings and the pyrazine ring are 14.66 (8) and 49.76 (12)°.

## Related literature

For background to organic light-emitting diodes based on phospho­rescent complexes, see: Baldo *et al.* (1998 ▶, 2000 ▶). For the synthesis of the title compound, see: Ge *et al.* (2009 ▶).
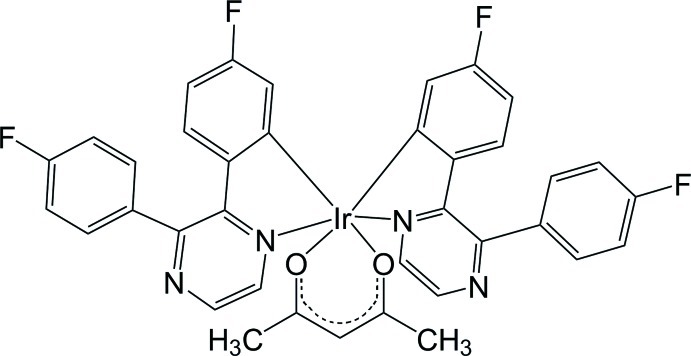


## Experimental

### 

#### Crystal data

[Ir(C_16_H_9_F_2_N_2_)_2_(C_5_H_7_O_2_)]
*M* *_r_* = 825.83
Monoclinic, 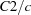

*a* = 21.030 (4) Å
*b* = 10.010 (2) Å
*c* = 16.118 (3) Å
β = 105.58 (3)°
*V* = 3268.3 (12) Å^3^
*Z* = 4
Mo *K*α radiation
μ = 4.15 mm^−1^
*T* = 293 K
0.40 × 0.28 × 0.18 mm

#### Data collection

Rigaku R-AXIS RAPID diffractometer
Absorption correction: multi-scan (*ABSCOR*; Higashi, 1995 ▶) *T*_min_ = 0.263, *T*_max_ = 0.470
14813 measured reflections
3714 independent reflections
3573 reflections with *I* > 2σ(*I*)
*R*_int_ = 0.074

#### Refinement

*R*[*F* ^2^ > 2σ(*F* ^2^)] = 0.032
*wR*(*F* ^2^) = 0.079
*S* = 1.03
3714 reflections
218 parameters
H-atom parameters constrained
Δρ_max_ = 2.15 e Å^−3^
Δρ_min_ = −2.10 e Å^−3^

### 

Data collection: *RAPID-AUTO* (Rigaku, 1998 ▶); cell refinement: *RAPID-AUTO*; data reduction: *CrystalStructure* (Rigaku/MSC, 2002 ▶); program(s) used to solve structure: *SHELXS97* (Sheldrick, 2008 ▶); program(s) used to refine structure: *SHELXL97* (Sheldrick, 2008 ▶); molecular graphics: *XP* in *SHELXTL* (Sheldrick, 2008 ▶); software used to prepare material for publication: *SHELXTL*.

## Supplementary Material

Crystal structure: contains datablock(s) global, I. DOI: 10.1107/S1600536812031546/hy2570sup1.cif

Structure factors: contains datablock(s) I. DOI: 10.1107/S1600536812031546/hy2570Isup2.hkl

Additional supplementary materials:  crystallographic information; 3D view; checkCIF report

## Figures and Tables

**Table 1:** Selected bond lengths (Å)

Ir—C6	1.987 (3)
Ir—N1	2.009 (3)
Ir—O1	2.153 (2)

**Fig. 1.**
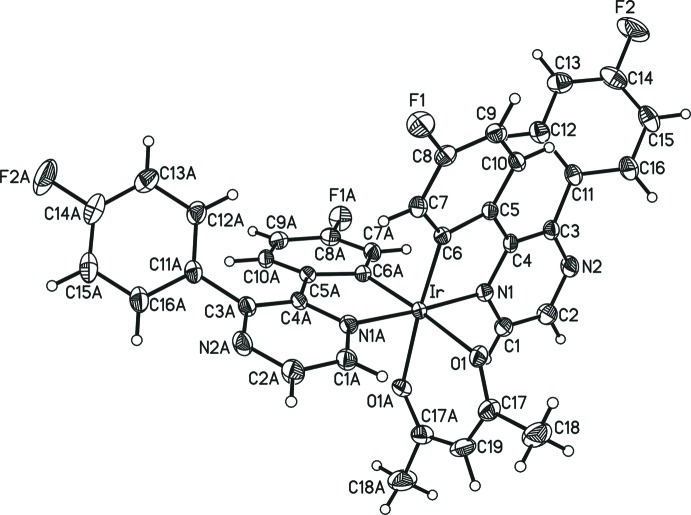
The molecular structure of the title complex, showing displacement ellipsoids at the 30% probability level. [Symmetry code: (A) -x, y, -z+1/2.]

## supplementary crystallographic information

### Comment

Much attention has been paid to the phosphorescent materials in recent years for their potential applications as highly efficient electroluminescent (EL) emitters in organic light-emitting devices (OLEDs), since the first demonstration of highly efficient phosphorescent OLEDs with a maximum EQE of 4% were reported by Baldo *et al.* (1998, 2000). Among these phosphorescent complexes, iridium cyclometalates often exhibit favorable photoproperties for OLEDs including short phosphorescent lifetimes, high quantum efficiencies and good stability. Ge *et al.* (2009) demonstrated a high efficiency yellow OLED using [Ir(dppf)_2_(acac)] [dppf = 2,3-di(4-fluorophenyl)pyrazine, acac = acetylacetone] as the dopant. In this work, we synthesized and investigated crystal structure of Ir(dppf)_2_(acac).

The mononuclear title iridium(III) complex (Fig. 1) has an approximately octahedral coordination geometry. The Ir^III^ ion is hexacoordinated by two C atoms and two N atoms from two C,N-bidentate dppf ligands, which exhibit *cis*-C,C and *trans*-N,N chelate dispositions, and two O atoms from one O,O-bidentate acac ligand. The Ir—C, Ir—N and Ir—O bond lengths are listed in Table 1. Due to steric interactions, the phenyl groups are not coplanar with the pyrazine group. The dihedral angles are 14.66 (8)° between the N1,N2/C1–C4 and C5–C10 rings and 49.76 (12)° between the N1,N2/C1–C4 and C11–C16 rings.

### Experimental

The title complex was obtained according to the procedure previously reported (Ge *et al.*, 2009). Orange crystals of the title complex suitable for X-ray structure analysis were grown from a mixed solution of dichloromethane and ethanol (v/v, 1:3).

### Refinement

H atoms were placed in calculated positions and treated using a riding model, with C—H = 0.93 (aromatic) and 0.96 (CH_3_) Å and with *U*_iso_(H) = 1.2(1.5 for methyl)*U*_eq_(C). The highest residual electron density was found at 0.81 Å from Ir atom and the deepest hole at 1.01 Å from Ir atom.

### Crystal data

**Table:**

[Ir(C_16_H_9_F_2_N_2_)_2_(C_5_H_7_O_2_)]	*F*(000) = 1616.0
*M**_r_* = 825.83	*D*_x_ = 1.678 Mg m^−^^3^
Monoclinic, *C*2/*c*	Mo *K*α radiation, λ = 0.71073 Å
Hall symbol: -C 2yc	Cell parameters from 3730 reflections
*a* = 21.030 (4) Å	θ = 3.3–27.4°
*b* = 10.010 (2) Å	µ = 4.15 mm^−^^1^
*c* = 16.118 (3) Å	*T* = 293 K
β = 105.58 (3)°	Cylindric, orange
*V* = 3268.3 (12) Å^3^	0.40 × 0.28 × 0.18 mm
*Z* = 4	

### Data collection

**Table:**

Rigaku R-AXIS RAPID diffractometer	3714 independent reflections
Radiation source: rotation anode	3573 reflections with *I* > 2σ(*I*)
Graphite monochromator	*R*_int_ = 0.074
ω scans	θ_max_ = 27.4°, θ_min_ = 3.3°
Absorption correction: multi-scan (*ABSCOR*; Higashi, 1995)	*h* = −27→27
*T*_min_ = 0.263, *T*_max_ = 0.470	*k* = −12→12
14813 measured reflections	*l* = −20→19

### Refinement

**Table:**

Refinement on *F*^2^	Primary atom site location: structure-invariant direct methods
Least-squares matrix: full	Secondary atom site location: difference Fourier map
*R*[*F*^2^ > 2σ(*F*^2^)] = 0.032	Hydrogen site location: inferred from neighbouring sites
*wR*(*F*^2^) = 0.079	H-atom parameters constrained
*S* = 1.03	*w* = 1/[σ^2^(*F*_o_^2^) + (0.0468*P*)^2^ + 2.1663*P*] where *P* = (*F*_o_^2^ + 2*F*_c_^2^)/3
3714 reflections	(Δ/σ)_max_ = 0.001
218 parameters	Δρ_max_ = 2.15 e Å^−^^3^
0 restraints	Δρ_min_ = −2.10 e Å^−^^3^

### Special details

**Table:**

Geometry. All e.s.d.'s (except the e.s.d. in the dihedral angle between two l.s. planes) are estimated using the full covariance matrix. The cell e.s.d.'s are taken into account individually in the estimation of e.s.d.'s in distances, angles and torsion angles; correlations between e.s.d.'s in cell parameters are only used when they are defined by crystal symmetry. An approximate (isotropic) treatment of cell e.s.d.'s is used for estimating e.s.d.'s involving l.s. planes.
Refinement. Refinement of *F*^2^ against ALL reflections. The weighted *R*-factor *wR* and goodness of fit *S* are based on *F*^2^, conventional *R*-factors *R* are based on *F*, with *F* set to zero for negative *F*^2^. The threshold expression of *F*^2^ > σ(*F*^2^) is used only for calculating *R*-factors(gt) *etc*. and is not relevant to the choice of reflections for refinement. *R*-factors based on *F*^2^ are statistically about twice as large as those based on *F*, and *R*- factors based on ALL data will be even larger.

### Fractional atomic coordinates and isotropic or equivalent isotropic displacement parameters (Å^2^)

**Table:**

	*x*	*y*	*z*	*U*_iso_*/*U*_eq_	
Ir	0.0000	0.399077 (13)	0.2500	0.02715 (8)	
N1	−0.08587 (17)	0.4046 (2)	0.1576 (2)	0.0322 (6)	
N2	−0.20277 (17)	0.4187 (3)	0.0278 (2)	0.0438 (8)	
F1	0.15157 (10)	0.7392 (3)	0.12655 (16)	0.0565 (6)	
F2	−0.2418 (2)	0.9357 (4)	−0.1927 (3)	0.0993 (12)	
C1	−0.13642 (17)	0.3191 (3)	0.1545 (2)	0.0403 (7)	
H2A	−0.1330	0.2571	0.1985	0.048*	
C2	−0.19232 (18)	0.3227 (4)	0.0876 (3)	0.0491 (9)	
H1A	−0.2240	0.2565	0.0836	0.059*	
C3	−0.15473 (15)	0.5090 (3)	0.03242 (19)	0.0340 (6)	
C4	−0.09249 (14)	0.4969 (3)	0.09367 (18)	0.0294 (6)	
C5	−0.03044 (15)	0.5686 (3)	0.0989 (2)	0.0287 (6)	
C6	0.02113 (15)	0.5378 (3)	0.1734 (2)	0.0286 (6)	
C7	0.0829 (2)	0.5995 (3)	0.1821 (3)	0.0360 (8)	
H25A	0.1175	0.5854	0.2311	0.043*	
C8	0.09109 (16)	0.6806 (3)	0.1172 (2)	0.0377 (7)	
C9	0.04288 (16)	0.7039 (3)	0.0422 (2)	0.0362 (6)	
H16A	0.0514	0.7559	−0.0014	0.043*	
C10	−0.01820 (16)	0.6482 (3)	0.0333 (2)	0.0326 (6)	
H15A	−0.0518	0.6634	−0.0167	0.039*	
C11	−0.17425 (18)	0.6233 (4)	−0.0285 (3)	0.0375 (8)	
C12	−0.16476 (17)	0.7527 (4)	0.0010 (2)	0.0439 (8)	
H14A	−0.1429	0.7691	0.0583	0.053*	
C13	−0.1875 (2)	0.8589 (5)	−0.0543 (3)	0.0549 (10)	
H12A	−0.1811	0.9467	−0.0350	0.066*	
C14	−0.2196 (2)	0.8303 (5)	−0.1381 (3)	0.0632 (12)	
C15	−0.2303 (2)	0.7040 (5)	−0.1699 (3)	0.0624 (12)	
H13A	−0.2524	0.6887	−0.2273	0.075*	
C16	−0.2074 (2)	0.5988 (4)	−0.1142 (3)	0.0492 (11)	
H3A	−0.2141	0.5114	−0.1341	0.059*	
C17	0.0240 (2)	0.1210 (4)	0.1865 (3)	0.0475 (10)	
C18	0.0470 (3)	0.0333 (5)	0.1240 (4)	0.0766 (15)	
H21A	0.0621	0.0883	0.0844	0.115*	
H21B	0.0110	−0.0214	0.0928	0.115*	
H21C	0.0825	−0.0227	0.1552	0.115*	
C19	0.0000	0.0599 (6)	0.2500	0.0603 (16)	
H22A	0.0000	−0.0330	0.2500	0.072*	
O1	0.02969 (13)	0.2442 (3)	0.17557 (16)	0.0398 (5)	

### Atomic displacement parameters (Å^2^)

**Table:**

	*U*^11^	*U*^22^	*U*^33^	*U*^12^	*U*^13^	*U*^23^
Ir	0.02894 (11)	0.02803 (11)	0.02172 (11)	0.000	0.00204 (7)	0.000
N1	0.0352 (15)	0.0325 (15)	0.0286 (14)	−0.0030 (9)	0.0080 (13)	−0.0008 (9)
N2	0.0350 (16)	0.0511 (18)	0.0377 (17)	−0.0084 (12)	−0.0036 (14)	0.0000 (13)
F1	0.0401 (11)	0.0654 (15)	0.0638 (14)	−0.0176 (10)	0.0139 (11)	0.0071 (12)
F2	0.110 (3)	0.087 (2)	0.088 (3)	0.024 (2)	0.003 (2)	0.051 (2)
C1	0.0406 (17)	0.0391 (18)	0.0378 (16)	−0.0104 (13)	0.0042 (14)	0.0021 (13)
C2	0.0415 (18)	0.050 (2)	0.049 (2)	−0.0167 (15)	−0.0011 (17)	0.0013 (16)
C3	0.0318 (14)	0.0389 (16)	0.0276 (14)	0.0016 (12)	0.0017 (12)	−0.0027 (13)
C4	0.0318 (13)	0.0324 (14)	0.0222 (12)	0.0016 (11)	0.0039 (11)	−0.0037 (11)
C5	0.0310 (14)	0.0284 (13)	0.0251 (14)	0.0024 (12)	0.0050 (12)	−0.0018 (12)
C6	0.0295 (14)	0.0272 (15)	0.0271 (14)	0.0021 (11)	0.0040 (12)	−0.0020 (11)
C7	0.0309 (17)	0.040 (2)	0.0333 (19)	−0.0029 (11)	0.0015 (16)	−0.0009 (11)
C8	0.0329 (15)	0.0367 (17)	0.0448 (17)	−0.0054 (12)	0.0129 (14)	−0.0034 (14)
C9	0.0424 (16)	0.0335 (15)	0.0364 (16)	−0.0001 (13)	0.0172 (14)	0.0005 (13)
C10	0.0358 (15)	0.0352 (16)	0.0258 (14)	0.0043 (13)	0.0062 (12)	0.0009 (13)
C11	0.0304 (16)	0.0477 (19)	0.0318 (18)	0.0044 (13)	0.0036 (14)	0.0022 (14)
C12	0.0367 (17)	0.050 (2)	0.0428 (19)	0.0119 (15)	0.0071 (15)	0.0030 (16)
C13	0.047 (2)	0.047 (2)	0.069 (3)	0.0087 (18)	0.013 (2)	0.009 (2)
C14	0.057 (2)	0.071 (3)	0.059 (3)	0.019 (2)	0.010 (2)	0.029 (2)
C15	0.058 (2)	0.081 (3)	0.0391 (19)	0.012 (2)	−0.0029 (19)	0.012 (2)
C16	0.044 (2)	0.061 (3)	0.037 (2)	0.0067 (14)	0.001 (2)	0.0027 (14)
C17	0.055 (2)	0.0367 (19)	0.050 (2)	0.0042 (15)	0.012 (2)	−0.0064 (16)
C18	0.118 (4)	0.046 (3)	0.076 (3)	0.009 (3)	0.044 (3)	−0.011 (2)
C19	0.094 (5)	0.026 (2)	0.065 (4)	0.000	0.029 (4)	0.000
O1	0.0494 (14)	0.0366 (13)	0.0322 (12)	0.0021 (10)	0.0087 (11)	−0.0069 (10)

### Geometric parameters (Å, º)

**Table:**

Ir—C6	1.987 (3)	C9—C10	1.372 (5)
Ir—N1	2.009 (3)	C9—H16A	0.9300
Ir—O1	2.153 (2)	C10—H15A	0.9300
N1—C4	1.363 (4)	C11—C12	1.375 (6)
N1—C1	1.356 (4)	C11—C16	1.392 (6)
N2—C3	1.342 (5)	C12—C13	1.387 (6)
N2—C2	1.337 (5)	C12—H14A	0.9300
F1—C8	1.372 (4)	C13—C14	1.369 (7)
F2—C14	1.373 (5)	C13—H12A	0.9300
C1—C2	1.366 (5)	C14—C15	1.360 (7)
C1—H2A	0.9300	C15—C16	1.384 (6)
C2—H1A	0.9300	C15—H13A	0.9300
C3—C4	1.417 (4)	C16—H3A	0.9300
C3—C11	1.492 (5)	C17—O1	1.256 (5)
C4—C5	1.472 (4)	C17—C19	1.399 (6)
C5—C10	1.403 (5)	C17—C18	1.509 (6)
C5—C6	1.418 (4)	C18—H21A	0.9600
C6—C7	1.411 (5)	C18—H21B	0.9600
C7—C8	1.371 (5)	C18—H21C	0.9600
C7—H25A	0.9300	C19—C17^i^	1.399 (6)
C8—C9	1.374 (5)	C19—H22A	0.9300
			
C6^i^—Ir—C6	91.30 (18)	F1—C8—C7	118.2 (3)
C6^i^—Ir—N1	97.75 (12)	F1—C8—C9	117.9 (3)
C6—Ir—N1	80.01 (12)	C7—C8—C9	123.9 (3)
C6^i^—Ir—N1^i^	80.01 (12)	C10—C9—C8	118.2 (3)
C6—Ir—N1^i^	97.75 (12)	C10—C9—H16A	120.9
N1—Ir—N1^i^	176.82 (13)	C8—C9—H16A	120.9
C6^i^—Ir—O1	175.30 (9)	C9—C10—C5	120.5 (3)
C6—Ir—O1	90.58 (13)	C9—C10—H15A	119.7
N1—Ir—O1	86.82 (11)	C5—C10—H15A	119.7
N1^i^—Ir—O1	95.48 (11)	C12—C11—C16	119.7 (3)
C6^i^—Ir—O1^i^	90.58 (13)	C12—C11—C3	120.5 (3)
C6—Ir—O1^i^	175.30 (9)	C16—C11—C3	119.6 (3)
N1—Ir—O1^i^	95.48 (11)	C11—C12—C13	120.5 (4)
N1^i^—Ir—O1^i^	86.82 (11)	C11—C12—H14A	119.8
O1—Ir—O1^i^	87.88 (15)	C13—C12—H14A	119.8
C4—N1—C1	118.7 (3)	C14—C13—C12	117.9 (5)
C4—N1—Ir	117.9 (2)	C14—C13—H12A	121.1
C1—N1—Ir	123.3 (2)	C12—C13—H12A	121.1
C3—N2—C2	117.9 (3)	C13—C14—C15	123.6 (4)
N1—C1—C2	120.8 (3)	C13—C14—F2	117.7 (5)
N1—C1—H2A	119.6	C15—C14—F2	118.7 (5)
C2—C1—H2A	119.6	C14—C15—C16	118.0 (4)
N2—C2—C1	121.9 (3)	C14—C15—H13A	121.0
N2—C2—H1A	119.0	C16—C15—H13A	121.0
C1—C2—H1A	119.0	C15—C16—C11	120.3 (4)
N2—C3—C4	121.5 (3)	C15—C16—H3A	119.9
N2—C3—C11	114.2 (3)	C11—C16—H3A	119.9
C4—C3—C11	124.3 (3)	O1—C17—C19	126.8 (4)
N1—C4—C3	118.2 (3)	O1—C17—C18	114.7 (4)
N1—C4—C5	112.2 (3)	C19—C17—C18	118.5 (4)
C3—C4—C5	129.6 (3)	C17—C18—H21A	109.5
C10—C5—C6	120.5 (3)	C17—C18—H21B	109.5
C10—C5—C4	125.0 (3)	H21A—C18—H21B	109.5
C6—C5—C4	114.0 (3)	C17—C18—H21C	109.5
C7—C6—C5	117.6 (3)	H21A—C18—H21C	109.5
C7—C6—Ir	126.7 (2)	H21B—C18—H21C	109.5
C5—C6—Ir	115.5 (2)	C17^i^—C19—C17	128.1 (6)
C8—C7—C6	118.9 (3)	C17^i^—C19—H22A	115.9
C8—C7—H25A	120.6	C17—C19—H22A	115.9
C6—C7—H25A	120.6	C17—O1—Ir	125.2 (3)

Symmetry code: (i) −*x*, *y*, −*z*+1/2.
